# Rediscovering two *Isoetes* species in the Brazilian Amazon and Cerrado after 167 years

**DOI:** 10.3897/phytokeys.135.46624

**Published:** 2019-12-05

**Authors:** Jovani B. S. Pereira, Ana Maria Giulietti, Vali J. Pott, Maurício T. C. Watanabe

**Affiliations:** 1 Instituto Tecnológico Vale, Rua Boaventura da Silva, 955, 66055-090, Belém, PA, Brazil Instituto Tecnológico Vale Belém Brazil; 2 Depto, Botânica da Universidade Estadual de Feira de Santana, Feira de Santana-BA, Brazil Universidade Estadual de Feira de Santana Feira de Santana Brazil; 3 Universidade Federal de Mato Grosso do Sul, Campo Grande – MS, Brazil Universidade Federal de Mato Grosso do Sul Campo Grande Brazil

**Keywords:** Aquatic plants, conservation status, endemic species, fieldworks, Isoetaceae, taxonomy

## Abstract

*Isoetes amazonica* and *I. gardneriana* were the first two species of the genus to be collected from Brazil. *Isoetes amazonica* was gathered by Richard Spruce in the Amazon basin near Santarém in the state of Pará in 1850. *Isoetes gardneriana* was collected by George Gardner in the current Dianópolis in Tocantins State in 1843. Despite being known for a long time by botanists, these species have not been recollected since then, which raised questions about their taxonomic recognition, current distribution ranges and conservation status. Fieldwork efforts led to the rediscovery of *I. amazonica* and *I. gardneriana* after 167 years. These collections enrich our understanding of their habitats and morphologies. We provide here re-descriptions for these species. Based on IUCN criteria, *Isoetes amazonica* and *I. gardneriana* should be assigned as data deficient (DD) and endangered (EN), respectively. The rediscovery of these species raises hopes that other areas in Amazon and Cerrado biomes harbour *I. amazonica* and *I. gardneriana*, respectively. This study will serve as a basis towards the conservation of these species.

## Introduction

Brazil presents the greatest diversity of plants in the world (Forzza et al. 2012), which partially reflects its large quantity of habitats. Particularly, habitats of its two largest biomes ‒ Amazon and Cerrado ‒ are undergoing a rapid reduction due to deforestation and large scale agriculture, including soybean and cattle farming and construction of hydroelectric dams (Laurance et al. 2000; Carvalho et al. 2009). At the same time, these areas remain largely unexplored botanically (Sousa-Baena et al. 2014), which raises conservation concerns about the numerous “lost plant species” that have been known only from type specimens.

The lycophyte genus *Isoetes* L. is globally distributed with an estimated 250 species (Troia et al. 2016), 22 of them being endemic to Brazil (Prado et al. 2015). The genus is frequently overlooked by botanists due to its resemblance to grasses or sedges (Taylor and Hickey 1992) and due to its aquatic habitat occurring semi- to fully submerged up to 6‒7 m deep in water (Middelboe and Markager 1997). As a result, many species are known only from type specimens (e.g. Pereira et al. 2016, 2017; Hickey et al. 2009).

*Isoetes amazonica* A. Braun and *I. gardneriana* Kunze ex A. Braun were the first two *Isoetes* species to be collected and described from Brazil. *Isoetes amazonica* was first collected by Richard Spruce in September 1850 from inundated shores of the Tapajós river near Santarém municipality in the state of Pará (Kuhn 1884). *Isoetes gardneriana* was first found by George Gardner (1849: 236) in 1843 in a marsh by the side of the river Preto, Mission of Duro, in the state of Goiás (currently on the border between Tocantins and Bahia in the municipalities of Dianópolis and Formosa do Rio Preto, respectively). *Isoetes amazonica* was published in 1880, *I. gardneriana* in 1862 (see Troia et al. 2016) and further information about them was compiled in the “*Flora Brasiliensis*” of Martius by Kuhn (1884). Despite having been collected and known for a long time, *I. amazonica* and *I. gardneriana* have not been recollected for 167 years. Our lack of knowledge about these species raises questions about their taxonomic recognition, current distribution ranges and their conservation status.

Motivated by these issues, we embarked on an attempt to rediscover these species in both the type localities and other similar environments in Amazon basin and Brazilian Cerrado.

## Material and methods

For *Isoetes amazonica*, fieldwork was carried out along both banks of the Tapajós river, near the district Alter do Chão, municipality of Santarém, in the state of Pará, Brazil, in September 2016 and July 2017. For *Isoetes gardneriana*, fieldwork efforts were carried out along the margins of the Preto river in Formosa do Rio Preto (Bahia) in January 2018. Additional efforts to find this species took place in other Brazilian Cerrado areas: Ondas river, Barreiras (Bahia) ‒ ca. 200 km away from the type location ‒ in January 2018; Parque Nacional Serra da Mesa (Maranhão) ‒ 500 km away from the type location ‒ in November 2017; Parque Nacional da Serra do Cipó ‒ 900 km away from the type location ‒ in June 2018; Fazenda Modelo, Campo Experimental da Embrapa, Terenos (Mato Grosso do Sul) ‒ 1200 km away from the type location ‒ in November 2017.

Besides field trips, specimens from the following herbaria were consulted to check for previous records of these species (acronyms following Thiers 2018): CGMS, MG, RB and UPCB (Brazil); B, E, M, HBG, P and K (Europe). These materials were compared to type specimens of *I. amazonica* (Spruce 1081, K [K000574506]) and *I. gardneriana* (Gardner 3563, B and E [E00429095]).

We checked the total monthly precipitation and average monthly maximum and minimum temperatures of the environments of these species’ localities to understand the influence of both flooding and drought in their habitats and life forms. For *I. amazonica*, the climatic data were collected from the meteorological station located in Belterra in the state of Pará and made available by “Instituto Nacional de Meteorologia” (INMET 2019). The climatic data for *I. gardneriana* were obtained from Campo Grande in the state of Mato Grosso do Sul (MS) and made available by the “Centro de Monitoramento do Tempo e Clima, MS” (CEMTEC/MS 2019).

Habitat, life form, colour, size and ornamentation of the mega- and microspores, the proportion of the sporangium wall covered by the velum and the sporangial wall colouration were used in the identification of the species. The megaspores and microspores were analysed using scanning electron microscopy (SEM). Images of the spores were made by transferring the spores to aluminium stubs coated with a carbon adhesive. The stubs were then coated with gold-palladium-alloy in a sputter-coater for 180 s and then digitally imaged using a Zeiss SIGMA VP.

Since megaspore ornamentations are essential for the correct species identification, the absence of detailed images of spores during the determination process may have potentially led to the name *I. gardneriana* being misused for several collections of *I. panamensis* Maxon & C.V.Morton *sensu lato.* We consulted these materials to check whether the identification was correct or not in these cases. Amongst these materials were collections from: Paraguay in 1878 (Balansa 3294, P [P00170381, P00573953, P04459456]); municipality of Barreiras in Bahia, Brazil, in 1971 (Irwin 31615, P [P01591973]); an area next to type location of *I. gardneriana* in the municipality of Formosa do Rio Preto in Bahia, Brazil, in 2015 (Labiak 5783, UPCB with duplicates in NY [NY2697584]). In this step, megaspores of these materials were removed, images were taken using SEM and then compared with the type of *I. gardneriana*. We used both qualitative and quantitative characters to identify the species. The terminology used for the description of the spores follows that of Punt et al. (2007), with some modification using Pereira et al. (2016). Boxplots of the megaspore macro-ornamentation projects were generated using an R script (v. 3.0.2; R Core Team 2013).

## Results

### Rediscovering *I. amazonica* after 167 years and re-description of the species

#### Isoetes amazonica

Taxon classificationPlantaeIsoetalesIsoetaceae

A. Braun, J. Bot. 18: 109. 1880.

60F1455B-6783-5663-AE6E-519272C77725

##### Description.

Stems globose, 0.35‒0.7 cm wide, 3-lobate. Leaves 0.45‒1 mm wide at mid length, 4‒17 cm long, 9‒23 per individual, filiform, straight, ascending, apex acute; alae 0.8‒4.5 cm long, extending from the base 1/10 ‒ 1/4 of total leaf length, hyaline, membranaceous, attenuate. Subula present, olive green, trigonal. Labium present, 0.5‒0.7 × 0.9‒1.1 mm long, cordate. Ligule 2.5‒3 × 1‒1.2 mm, hyaline, triangular. Velum 0.1‒0.2 mm along the lateral edges of the sporangium, rudimentary. Sclerified phyllopodia absent. Sporangium at the base of the leaf, 2‒2.5 × 1.8‒2.5mm, elliptic, hyaline, light brown, brown dots present or absent. Megaspores white, 420‒512 (‒590) µm in equatorial diameter (average = 490 µm), trilete; laesurae as wide as high or higher than wide, 40‒53 × 35‒47 µm; proximal surface verrucate, projections 24‒41.4 × 24‒46 µm; equatorial ridges arched, slightly sinuous; distal surface verrucate, macroscuptural projections 25‒45 × 25‒46 µm. Microspores 28‒32 µm long (average = 30 µm), proximal surface echinate, distal surface sparsely echinate.

##### Type.

Brazil. Province of Pará: inundated places near Santarém, Sept 1850, Spruce 1081, (holotype: B! [B200107121]; isotype: K! [K000574506], P! [P00573942; P00573943]).

##### Remarks.

**Figure 1.**
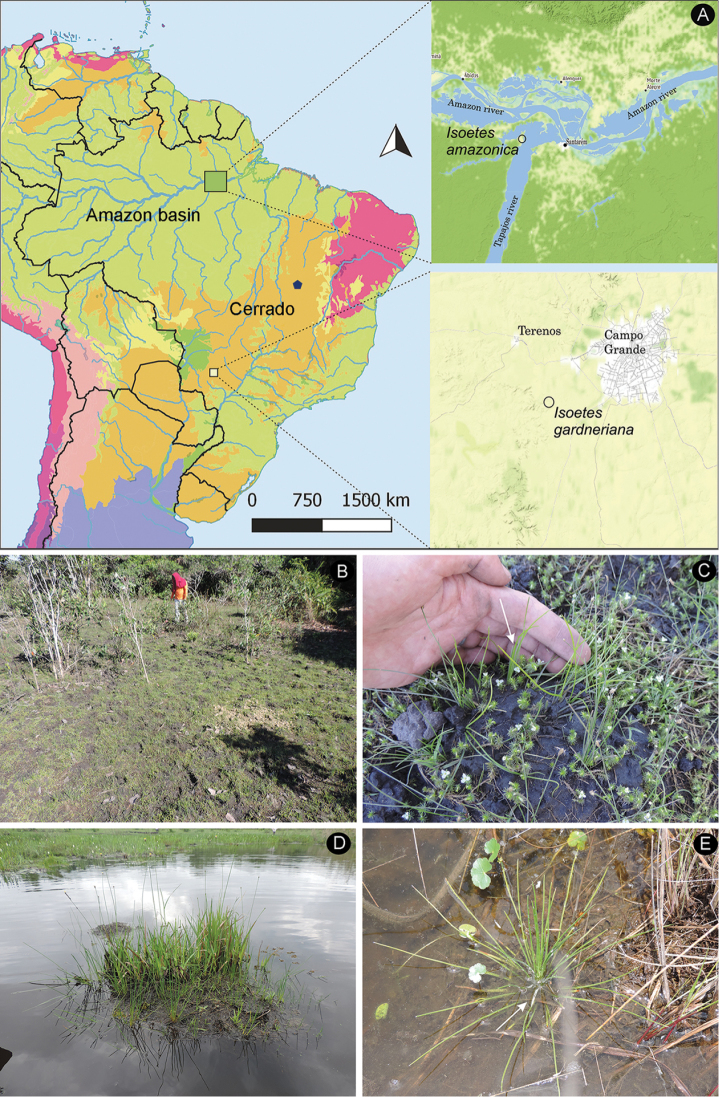
Geographic distribution, habit and habitat of *Isoetes amazonica* and *I. gardneriana***A** location where *Isoetes amazonica* and *I. gardneriana* were rediscovered in Brazil (type location of *I. gardneriana* in blue pentagon) **B–C***Isoetes amazonica* (*Pereira 1015*, MG): **B** habitat **C** habit **D–E***Isoetes gardneriana* (*Pereira 1028*, MG): **D** habitat **E** habit.

**Figure 2.**
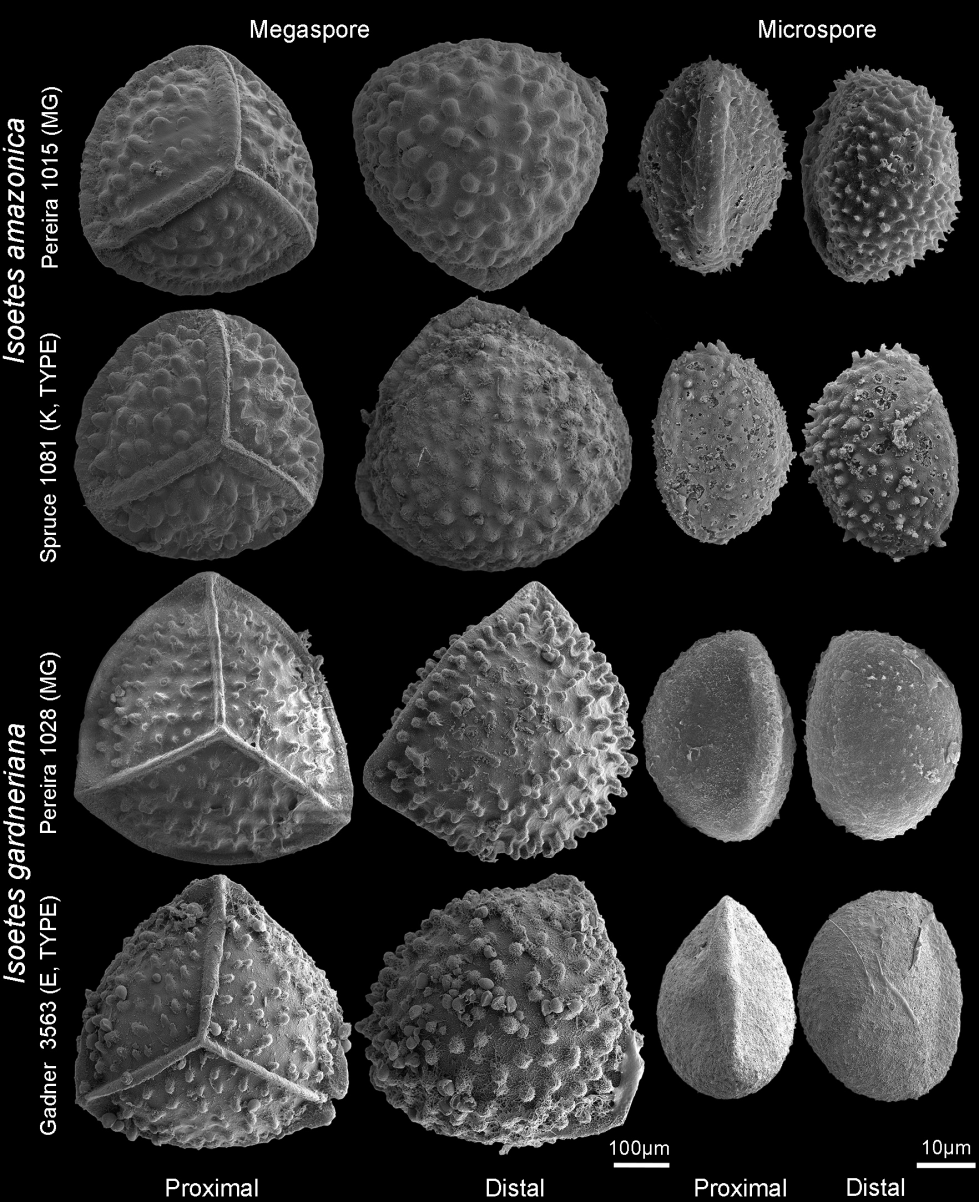
Mega- and microspores of *Isoetes amazonica* and *I. gardneriana*.

*Isoetes amazonica* was rediscovered at its type location in July 2017 after 167 years (Pereira 1015, MG). This species was found in a single area at approximately 2.5 km from the left bank of the Tapajós river at the geographical coordinates 2°24'15.15"S, 55°3'1.89"W (Figure 1A). This location was a marsh area between a flooded forest and cattle farming. The plants were found as terrestrials in wet clay and sandy soils (Fig. 1B, C). None of the individuals was completely submerged (but see discussion). The monthly precipitation was 42.4 mm and it was the lowest recorded value for July between the years of 2008 and 2017 (INMET 2019). The average monthly maximum and minimum temperatures were 32 and 22 °C, respectively (INMET 2019). This species occurred in association with other plant groups such as Cyperaceae, Poaceae, Mayacaceae and Eriocaulaceae. The newly rediscovered population showed typical characteristics of *I. amazonica*, such as 10‒20 leaves per individual, ascending leaves, rudimentary velum, hyaline sporangia with or without brown spots, verrucate megaspores with 470‒590 µm diameter, sparsely echinate microspores 28‒32 µm long (Fig. 2).

### New record of *I. gardneriana* at about 1200 km away from its type location and re-description of the species

#### Isoetes gardneriana

Taxon classificationPlantaeIsoetalesIsoetaceae

Kunze ex A. Braun, Verh. Bot. Vereins Prov. Brandenburg 4: 330. 1862.

978D4FE7-13F7-5BBE-8CF9-D39EEB8AB6ED

##### Description.

Stems globose, 2.5‒4 cm wide, 3 or 4-lobate. Leaves 1.0‒1.8 mm wide at mid length, 32‒45 cm long, 30‒90 per individual, linear, straight, ascending, apex acute; alae 7‒15 cm long, extending from the base 1/5‒2/5 of total leaf length, hyaline or light brown, chartaceous, attenuate. Subula present, olive green, trigonal. Labium present, 2.5‒3.5 × 4‒6 mm, cordate. Ligule not observed. Velum > 0.4 mm along the lateral edges of the sporangium, rudimentary. Sclerified phyllopodia absent. Sporangium at the base of the leaf, 8‒18 × 4.3‒7 mm, oblong, hyaline, brown dots absent. Megaspores grey, 490‒650 µm in equatorial diameter (average = 540 µm), trilete; laesures higher than wide, 40‒50 × 11‒16 µm; proximal surface tuberculate, macrosculptural projections 20‒39 × 13‒24 µm; equatorial ridges arched, straight; distal surface tuberculate, projections 24‒44 × 17‒34 µm. Microspores 33‒40 µm long (average = 37 µm), proximal and distal surface smooth or sparsely microechinate.

##### Type.

Brazil. Province of Goyaz: Missiones Duro, Sept 1841, Gardner 3563, (holotype: B! [B200107577]; isotype: BM [BM000097912,] E! [E00429095], K! [K000574505]).

##### Remarks.

Despite our intensive fieldwork efforts in the Brazilian Cerrado, *I. gardneriana* was only rediscovered in Terenos in the state of Mato Grosso do Sul at the geographical coordinates 20°33'32"S, 54°47'23"W. This area is located at about 1200 km away from its type location (Fig. 1A). It was collected there by both Vali Pott in September 2010 (Pott 11018, CGMS) and Jovani Pereira in November 2017 (Pereira 1028, MG) after 167 and 175 years, respectively. Although these records were far from the type location, habitat and morphology of this newly collected population are almost identical to the type.

*Isoetes gardneriana* was found in a pond along with *Rhynchospora corymbosa* (L.) Britton, *Pontederia cordata* L. and *Xyris* spp. (Fig. 1D). This pond occurred by the side of a “vereda”, which showed a clay and hydromorphic soil and an open vegetation physiognomy with the presence of numerous “buriti” palms (*Mauritia flexuosa* L.f.) that grow over a dense herbaceous stratum. The life form of *I. gardneriana* was of a partially submerged aquatic in September, although in November, plants were found both partially and totally submerged (Fig. 1E). The total monthly precipitations were 127 and 315.8 mm in September 2010 and November 2017, respectively (CEMTEC/MS 2019). The average monthly maximum and minimum temperatures were 32 and 22 °C, respectively (CEMTEC/MS 2019). The maximum and minimum temperature averages were 32.7 and 19.6 °C, respectively, in September 2010. In November 2017, the average monthly maximum and minimum temperatures were 30.7 and 20 °C, respectively (CEMTEC/MS 2019).

Morphologically, the individuals have ascending leaves, rudimentary vela, elliptic sporangia and 3-lobate corms (or more rarely 4). The megaspores are brown, sparsely verrucate, 490‒650 µm diameter (vs. 548‒615 µm), with knife-like laesurae (Fig. 2). The microspores are echinate, 33‒40 µm long (vs. 34‒38 µm) (Fig. 2).

**Figure 3.**
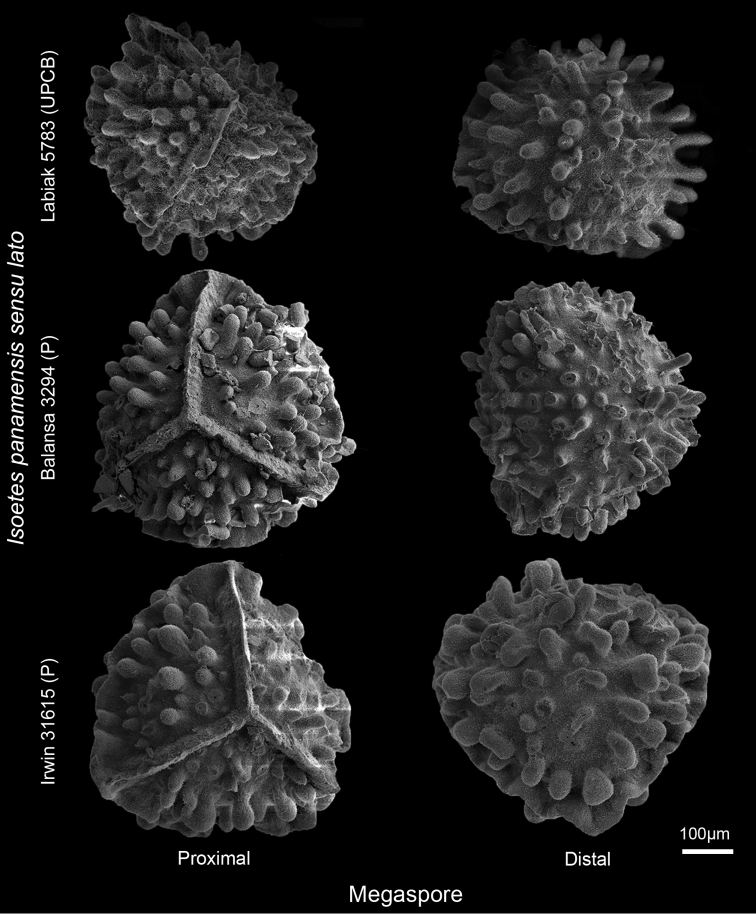
Megaspores of the variants of *Isoetes panamensissensu lato.*

**Figure 4.**
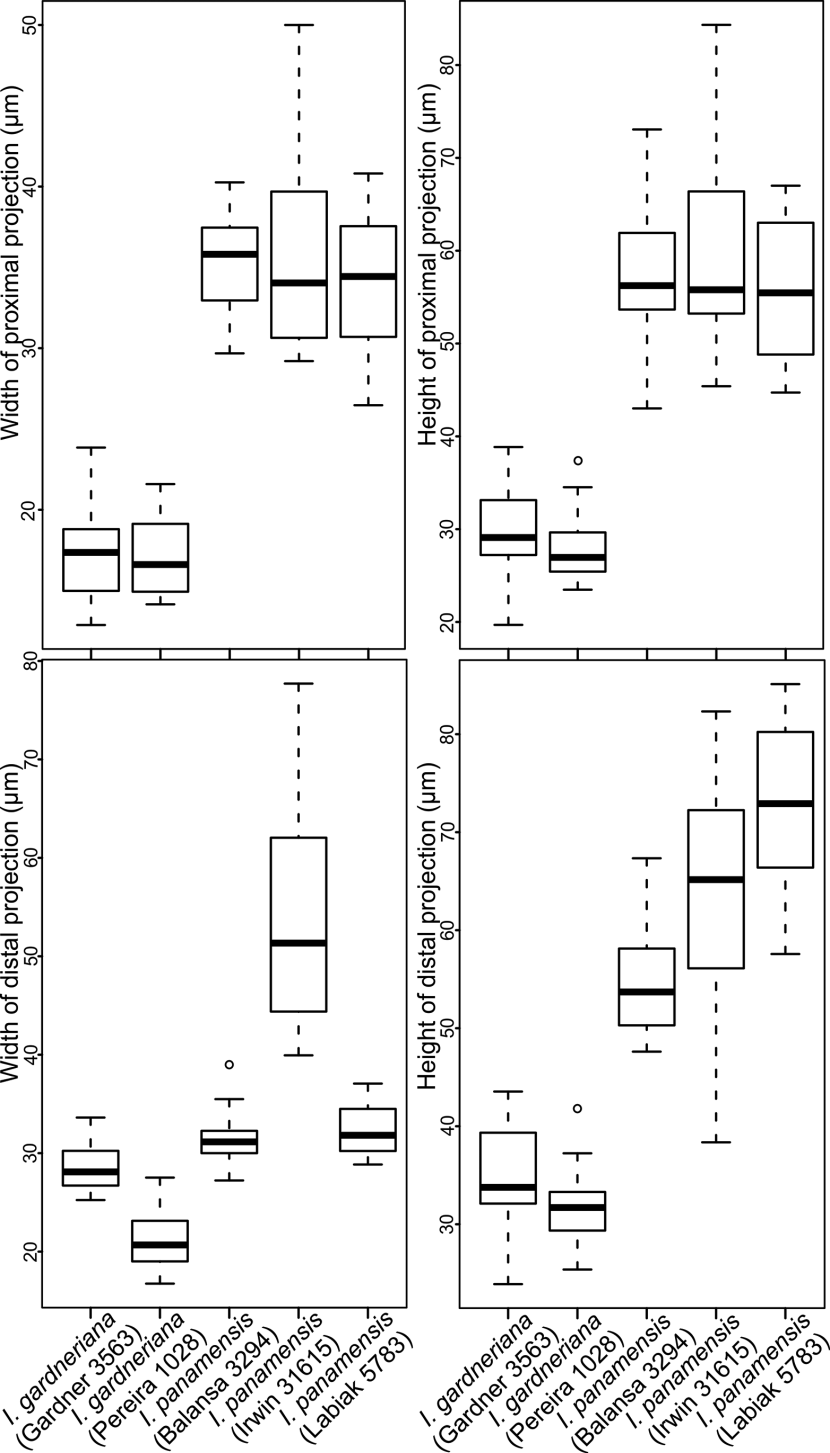
Boxplots showing quantitatively the variation in the size of the macro-ornamentation projections of the megaspores of *I. gardneriana* and *I. panamensissensu lato.* In the proximal surface, the projects of the macro-ornamentation are narrower and shorter in *I. gardneriana* than in *I. panamensiss.l.* In the distal surface, the macro-ornamentation projections are slightly narrower and considerably shorter in *I. gardneriana* than in *I. panamensis s.l..*

On the other hand, none of the analysed herbarium collections appeared (Balansa 3294, Irwin 31615 and Labiak 5783) to be *I. gardneriana*. The megaspores of these collections are both qualitatively and quantitatively distinct from the type of *I. gardneriana*. The Balansa and Labiak collections have baculate-tuberculate megaspores and Irwin’s collection revealed baculate-clavate megaspores (Fig. 3), which confirm that these materials represent variants of *I. panamensiss.l.* Additionally, the macro-ornamentation projections of the megaspores of *I. gardneriana* are, in general, narrower and shorter than those found in *I. panamensiss.l.* (Fig. 4).

## Discussion

Although fieldwork investigation is fundamental to improve our understanding about how human impacts on biological systems can be recognised, mitigated or averted, fieldwork has considerably decreased in the past decades with negative implications for global biodiversity conservation (Ríos-Saldaña et al. 2018). The rediscovering of these species was only possible due to intense fieldwork; otherwise, they would have remained little known to science.

Both proper habitat and taxonomic identification of species are the first steps towards conserving biodiversity. Amongst the aquatic macrophytes, *Isoetes* is one of the most threatened groups (Murphy et al. 2019). However, difficulties related to finding species in the field, identifying them morphologically and, consequently, establishing their geographical distribution, hamper efforts to assess their current conservation status. *Isoetes amazonica* and *I. gardneriana* were known only from their type materials collected 167 years ago, which raised questions about their current occurrences and morphological distinction. Our rediscoveries provide a basis for a better understanding of the distribution and taxonomy of these species, which will help develop a plan to conserve these plants.

Even though *I. amazonica* was collected only during the dry season, we can make inferences about its habitat conditions and life forms during the year, using climatic data (see INMET 2019). *Isoetes amazonica* was collected as a terrestrial at the beginning of the dry season in July. However, its life form may oscillate between terrestrial and completely aquatic due to the alternating flooding and drought conditions in the Amazon basin during the year (see Marengo and Espinoza 2016). Additionally, during the driest and hottest period in August-November, its habitat may entirely dry out and this species may lose its leaves due to the combination of low precipitation, decreasing of the water table above the surface and high temperature. On the other hand, during the peak of the rainy season in March, its habitat is flooded and *I. amazonica* may become a completely submerged aquatic. Similarly, *I. gardneriana* occurs in an area which undergoes dry and rainy seasons (see CEMTEC/MS 2019 for climatic data). However, *I. gardneriana* grows in the deepest part of a small pond just by the side of the “vereda” grassland, which stays waterlogged year-round and feeds this pond in the dry season (see Moreira et al. 2011). This factor leads its habitat to be marshy and flooded throughout the year and *I. gardneriana* may rarely be found as terrestrial.

Despite the importance of habitat data for species characterisation, they provide a limited amount of information for species distinction if two or more similar species occupy the same habitat and/or show morphological convergence due to habitat adaptation (e.g. Taylor and Hickey 1992; Jiménez-Mejías et al. 2017). Both *Isoetes gardneriana* and *I. panamensiss.l.* are found in areas of “veredas” in Cerrado, which partially contribute to taxonomic difficulties involving these two species. However, they can be distinguished by qualitative and quantitative characters of megaspores.

Additionally, an *Isoetes* population from Itaparica lake in Xique-Xique (Bahia State) in north-eastern Brazil was tentatively identified as *I. amazonica* (Harley 19109, K). However, despite its resemblance to *I. amazonica* by size of megaspores and number and size of leaves, the presence of brown sporangium (vs. hyaline) and its occurrence in Caatinga (vs. Amazon) leads us to believe that this population is either a variant of *I. luetzelburgii* U. Weber or an undescribed species.

The geographical distribution of the species is crucial in assessing their conservation status (IUCN 2016). In *Isoetes*, the proportion of species with narrow-range distributions is remarkably high (Prado et al. 2015). The same extreme restricted distribution patterns are also found in several other aquatic macrophytes, such as Podostemaceae, Araceae, especially *Cryptocoryne* spp., Cyperaceae and Eriocaulaceae (Murphy et al. 2019). However, in several cases, it appears unclear whether this pattern occurs due to endemism (driven by biological factors) or collection deficiency. Although more fieldwork efforts are needed to address this question in *I. amazonica*, this study revealed that *I. gardneriana* shows a much wider distribution than previously known.

*Isoetes amazonica* is currently known from a single locality next to a cattle farm and, thus, it is prone to the effects of human activities within a short time. However, given its potential occurrence in other areas in the Amazon basin and the lack of current knowledge about its distribution range, *I. amazonica* should be assessed as data deficient (DD), according to IUCN criteria (IUCN 2016). On the other hand, *I. gardneriana* – which is endemic to Cerrado – is clearly undergoing a population size reduction due to the loss of suitable habitats. The agri-business expansion, infrastructure development, weak legal protection and limited conservation incentives have led to the loss of 46% of Cerrado native vegetation and, by 2050, Cerrado may lose up to 34% of its remaining area (Strassburg et al. 2017). This habitat reduction will have a direct impact on *I. gardneriana* and the population size of this species may likely substantially decrease in the next years. Thus, *I. gardneriana* should be assigned as endangered (EN), according to IUCN criteria (IUCN 2016).

In conclusion, the rediscovering of these species raises hopes that other areas in Amazon and Cerrado biomes still harbour *Isoetes amazonica* and *I. gardneriana*, respectively. We hope that these rediscoveries spark research towards a deeper understanding of the life history of *Isoetes* and provide information for any future efforts to protect *Isoetes amazonica* and *I. gardneriana* from extinction.

## Supplementary Material

XML Treatment for Isoetes amazonica

XML Treatment for Isoetes gardneriana

## References

[B1] Carvalho FM Marco P Ferreira LG (2009) The Cerrado into-pieces: Habitat fragmentation as a function of landscape use in the savannas of central Brazil. Biological Conservation 142(7): 1392–1403. 10.1016/j.biocon.2009.01.031

[B2] CEMTEC/MS (2019) Centro de Monitoramento do Tempo e Clima no estado do Mato Grosso do Sul, Brasil. http://www.cemtec.ms.gov.br/boletins-meteorologicos [accessed 10.06.2019]

[B3] Forzza RC Baumgratz JFA Bicudo CEM Canhos DAL Carvalho Jr AA Coelho MAN Costa AF Costa DP Hopkins MG Leitman PM Lohmann LG Lughadha EN Maia LC Martinelli G Menezes M Morim MP Peixoto AL Pirani JR Prado J Queiroz LP Souza S Souza VC Stehmann JR Sylvestre LS Walter BMT Zappi DC (2012) New Brazilian floristic list highlights conservation challenges. Bioscience 62(1): 39–45. 10.1525/bio.2012.62.1.8

[B4] Gardner G (1849) Travels in the Interior of Brazil, Principally Through the Northern Provinces, and the Gold and Diamond Districts, During the Years 1836–1841. Reeve Benham & Reeve, London, 428 pp.

[B5] Hickey RJ Macluf CC Link-Pérez M (2009) *Isoetes maxima*, a new species from Brazil. American Fern Journal 99(3): 194–199. 10.1640/0002-8444-99.3.194

[B6] INMET (2019) Instituto Nacional de Meteorologia, Brasil. http://www.inmet.gov.br/portal/index.php?r=bdmep/bdmep [accessed 4.08.2019]

[B7] IUCN (2016) The IUCN red list categories and criteria, version 3.1. IUCN Red List Unit, Gland, Switzerland and Cambridge, UK. http://www.iucnredlist.org/technical-documents/categories-and-criteria [accessed 4.02.2019]

[B8] Jiménez-Mejías P Benítez-Benítez C Fernández-Mazuecos M Martín-Bravo S (2017) Cut from the same cloth: The convergent evolution of dwarf morphotypes of the Carex flava group (Cyperaceae) in Circum-Mediterranean mountains. PLoS One 12(12): e0189769. 10.1371/journal.pone.0189769 PMC5744957 29281689

[B9] Kuhn FAM (1884) Isoetaceae. In: Martius CFP (Eds) Flora Brasiliensis v.1, part 2., 645–648. http://florabrasiliensis.cria.org.br/search?taxon_id=1403 [accessed 4.02.2019]

[B10] Laurance WF Vasconcelos HL Lovejoy TE (2000) Forest loss and fragmentation in the Amazon: Implications for wildlife conservation. Oryx 34(1): 39–45. 10.1046/j.1365-3008.2000.00094.x

[B11] Marengo JA Espinoza JC (2016) Extreme seasonal droughts and floods in Amazonia: Causes, trends and impacts. International Journal of Climatology 36(3): 1033–1050. 10.1002/joc.4420

[B12] Middelboe AL Markager S (1997) Depth limits and minimum light requirements of freshwater macrophytes. Freshwater Biology 37(3): 553–568. 10.1046/j.1365-2427.1997.00183.x

[B13] Moreira SM Pott A Pott VJ Damasceno GA (2011) Structure of pond vegetation in a vereda in the Brazilian Cerrado. Rodriguésia 62(4): 721–729. 10.1590/S2175-78602011000400002

[B14] Murphy K Efremov A Davidson TA Molina-Navarro E Fidanza K Crivelari TCB Chambers P Tapia JG Varandas SM Springuel I Kennedy M Mormul RP Dibble E Hofstra D Lukács BA Gebler D Baastrup-Spohr L Urrutia-Estrada J (2019) World distribution, diversity and endemism of aquatic macrophytes. Aquatic Botany 158: 103–127. 10.1016/j.aquabot.2019.06.006

[B15] Pereira JBS Salino A Arruda A Stützel T (2016) Two new species of *Isoetes* (Isoetaceae) from northern Brazil. Phytotaxa 272(2): 141–148. 10.11646/phytotaxa.272.2.5

[B16] Pereira JBS Stützel T Schulz C (2017) *Isoetes nana*, a new species from the coastal mountains of southeastern Brazil. PhytoKeys 89: 91–105. 10.3897/phytokeys.89.20171 PMC5672152 29118652

[B17] Prado J Sylvestre LS Labiak PH Windish PG Salino A Barros ICL Hirai RY Almeida TE Santiago ACP Kieling-Rubio MA Pereira AFN Ollgaard B Ramos CGV Mickel JT Dittrich VAO Mynssen CM Schwartsburd PB Condack JPS Pereira JBS Matos FB (2015) Diversity of ferns and lycophytes in Brazil. Rodriguésia 66(4): 1073–1083. 10.1590/2175-7860201566410

[B18] Punt W Hoen PP Blackmore S Nilsson S Le Thomas A (2007) Glossary of pollen and spore terminology. Review of Palaeobotany and Palynology 143(1–2): 1–81. 10.1016/j.revpalbo.2006.06.008

[B19] R Core Team (2013) A Language and Environment for Statistical Computing, R Foundation for Statistical Computing, Wien. http://www.R-project.org/ [accessed 10.08.2018]

[B20] Ríos-Saldaña CA Delibes-Mateos M Ferreira CC (2018) Are fieldwork studies being relegated to second place in conservation science? Global Ecology and Conservation 14: e00389. 10.1016/j.gecco.2018.e00389

[B21] Sousa-Baena MS Garcia LC Peterson AT Brotons L (2014) Completeness of digital accessible knowledge of the plants of Brazil and priorities for survey and inventory. Diversity & Distributions 20(4): 369–381. 10.1111/ddi.12136

[B22] Strassburg BBN Brooks T Feltran-Barbieri R Iribarrem A Crouzeilles R Loyola R Latawiec AE Oliveira Filho FJB Scaramuzza CAM Scarano FR Soares-Filho B Balmford A (2017) Moment of truth for the Cerrado hotspot. Nature Ecology & Evolution 1: 0099. 10.1038/s41559-017-0099 28812670

[B23] Taylor WC Hickey RJ (1992) Habitat, evolution, and speciation in *Isoetes* Annals of the Missouri Botanical Garden 79(3): 613–622. 10.2307/2399755

[B24] Thiers BM (2018) The World’s Herbaria 2017: A summary Report Based on Data from Index Herbariorum (2^nd^ ed.). The New York Botanical Garden Press, 19 pp. 10.3897/biss.2.26440

[B25] Troia A Pereira JBS Kim C Taylor WC (2016) The genus *Isoetes* (Isoetaceae): A provisional checklist of the accepted and unresolved taxa. Phytotaxa 277(2): 101–145. 10.11646/phytotaxa.277.2.1

